# Are alcoholism treatments effective? The Project MATCH data

**DOI:** 10.1186/1471-2458-5-75

**Published:** 2005-07-14

**Authors:** Robert B Cutler, David A Fishbain

**Affiliations:** 1 Department of Psychiatry and Behavioral Sciences, University of Miami School of Medicine, Miami, Florida, USA

## Abstract

**Background:**

Project MATCH was the largest and most expensive alcoholism treatment trial ever conducted. The results were disappointing. There were essentially no patient-treatment matches, and three very different treatments produced nearly identical outcomes. These results were interpreted post hoc as evidence that all three treatments were quite effective. We re-analyzed the data in order to estimate effectiveness in relation to quantity of treatment.

**Methods:**

This was a secondary analysis of data from a multisite clinical trial of alcohol dependent volunteers (N = 1726) who received outpatient psychosocial therapy. Analyses were confined to the primary outcome variables, percent days abstinent (PDA) and drinks per drinking day (DDD). Overall tests between treatment outcome and treatment quantity were conducted. Next, three specific groups were highlighted. One group consisted of those who dropped out immediately; the second were those who dropped out after receiving only one therapy session, and the third were those who attended 12 therapy sessions.

**Results:**

Overall, a median of only 3% of the drinking outcome at follow-up could be attributed to treatment. However this effect appeared to be present at week one before most of the treatment had been delivered. The zero treatment dropout group showed great improvement, achieving a mean of 72 percent days abstinent at follow-up. Effect size estimates showed that two-thirds to three-fourths of the improvement in the full treatment group was duplicated in the zero treatment group. Outcomes for the one session treatment group were worse than for the zero treatment group, suggesting a patient self selection effect. Nearly all the improvement in all groups had occurred by week one. The full treatment group had improved in PDA by 62% at week one, and the additional 11 therapy sessions added only another 4% improvement.

**Conclusion:**

The results suggest that current psychosocial treatments for alcoholism are not particularly effective. Untreated alcoholics in clinical trials show significant improvement. Most of the improvement which is interpreted as treatment effect is not due to treatment. Part of the remainder appears to be due to selection effects.

## Background

A fundamental belief of addiction treatment is that therapy is effective. Addiction counselors are encouraged to use methods that have been shown to be effective in high quality clinical trials [1]. Three of the best of those methods were selected for Project MATCH, a large multicenter US trial designed to match the most effective treatment to individual patient characteristics. The criteria used to select the three treatments used in MATCH included the following: demonstrated clinical effectiveness; applicability to existing treatment programs and client populations, and distinctiveness from each other [2].

Project MATCH took great care to assure that the therapy was of the highest quality. Therapy was manualized and the three manuals [3-5] were organized into specific treatment sessions. Cognitive Behavioral Therapy (CBT) focused on handling thoughts about alcohol, dealing with urges, refusing drinks, avoiding situations that might lead to relapse, etc. Motivational Enhancement Therapy (MET) provided structured feedback about alcohol-related problems, and attempted to motivate commitment to change, to increase individual responsibility, and to enlist personal resources. Twelve Step Facilitation (TSF) was based on principles of Alcoholics Anonymous and it introduced the first three steps of AA and promoted active participation in AA. Therapists were required to have a Masters degree or Certified Addiction Counselor degree, a commitment to the particular therapeutic approach that they would provide (i.e., CBT, MET, or TSF), and at least two years experience. Therapist training was centralized at the coordinating center using seminars, required two supervised training cases, and also included some ongoing supervision of all sessions. Therapists taped their sessions with clients, and the tapes were scored at the coordinating center.

In some ways, the results of the MATCH clinical trial were disappointing. At the time it was concluded, in the late 1990s, it was the one of the most expensive clinical trials ever undertaken, costing 27 million dollars; it was conducted by the most seasoned alcoholism professional investigators, and it was designed to validate the top "cutting-edge" findings which had accumulated the strongest experimental support. Some 504 hypotheses were tested [6]. The final results did not support the hypotheses. There were essentially no matches between the therapeutic treatments and the participants above the level of random probability [6]. An analysis of the problem suggested that too many Type I errors were being made in the alcoholism literature [7]. Type I errors typically occur when an inappropriately large number of statistical tests are performed.

In announcing the disappointing MATCH results, the director of the National Institute of Alcohol Abuse and Alcoholism stated "All three treatments evaluated in Project MATCH produced excellent overall outcomes" [8]. That position was given scientific weight most notably by William Miller and colleagues in their paper "How effective is alcoholism treatment in the United States?" [9]. They suggested that treatment is extremely effective by way of presenting the MATCH results, and results of other large multi-site alcoholism trials. Table 1 shows outcome data abstracted from that paper. In addition to MATCH, the studies consisted of an analysis conducted by the Rand Corporation of treatment programs around the U.S. [10], a study at Veteran's Hospitals that compared disulfiram (Antabuse) to placebo [11], a VA study comparing lithium to placebo [12], a study on relapse [13], and a study comparing 12-step to cognitive-behavioral treatment [14]. Table 1 shows that the MATCH outcomes were very similar to the outcomes of the other studies.

**Table 1:** Alcoholism treatment 12 month outcome data cited by Miller to suggest that treatment is effective.

Multi-center trial	Percent days abstinent	Drinks per drinking day
Lithium/Placebo	83	
Disulfiram/Placebo	84	
Relapse prevention	85	5
MATCH	77	5
12 Step/Cognitive		8
Rand		5
		
Mean	82	6

The effectiveness of psychosocial therapy for alcoholism is being challenged in a number of different ways. Evidence is accumulating that extensive therapy may be no more effective that brief intervention [1,15]. Brief interventions are minimal types of therapies that can consist of simple expressions of concern about drinking delivered by a MD in a hospital trauma unit. There is a growing literature on "natural recovery" showing that many, if not most, individuals with serious alcohol consumption problems are able to recover without treatment [16]. A recently published meta-analysis [17] reported a significant improvement in untreated alcoholics enrolled in clinical trials. And there have been a few published trials that have concluded that therapy is not particularly effective [18,19].

The present study reports analyses of some overlooked data from Project MATCH. The overall relationship between treatment quantity (number of treatment sessions attended) and drinking measures was analyzed. Next, primary outcome data of participants who dropped out of treatment before receiving any therapy was compared to that of participants who attended each and every session for the full 12 weeks of therapy. Additionally we identified one anomalous group consisting of those participants who attended only one session of therapy. The data from this unusual group provides additional clues that help in interpreting the findings.

## Methods

The MATCH Data set was made available to qualified researchers after the study had been completed. As an NIAAA committee member designing another large multi-center funded alcoholism study, the senior author of this study (RBC) obtained an official copy of the Project MATCH Public Data Set (V1.0) from the Coordinating Center in Farmington, Connecticut on August 24, 1998. The trial was conducted with approval from the appropriate ethics committees, with informed written consents, and the procedure was in compliance the Helsinki Declaration. This data set comprised some 256 variables on all 1726 participants in Project MATCH. Drinking data were summarized for each participant at pre-treatment, at weekly intervals for the 12 weeks of the trial, and at monthly intervals during the follow-up. Drinking data consisted of two measures, percent days abstinent (PDA) and drinks per drinking day (DDD). Participants were identified by which of the three treatments they received, Cognitive Behavioral (CBT), Motivation Enhancement (MET) or Twelve-Step (TSF). The number of treatment sessions each participant attended was also included. It is this variable that is the focus of this study.

The first step of the analysis was to compare our data to official published results. Pearson correlations were computed between number of treatment sessions attended with percent days abstinent and with drinks per drinking days at follow-up. We then computed correlations between number of sessions and the drinking variables during treatment. Both our analyses and previously published MATCH analyses substituted transformed scores for the drinking measures in order to normalize the data and thereby meet the assumptions of the statistical tests. The transformed scores were supplied in the data set. In this study we used the transformed scores for all statistical tests and the original drinking measures for display purposes.

In the next step the participants were categorized into groups based on the number of treatment sessions they attended, ranging from 0 to 12. Those alcoholics who signed up for the study but who never attended any sessions were coded zero. Those who were coded 12 attended every session and received either CBT or TSF but not MET. MET consisted of only 4 sessions. The outcome variables analyzed in this study are restricted to the two primary MATCH outcome variables.

Three groups were formed consisting of participants who attended either 0, 1 or all 12 treatment sessions. Chi square tests were used to test relationships between categorical variables. Repeated measures Manovas were used to test the differences between groups over all time points. Drinking outcome at follow-up was a mean of the entire follow-up period (from month 4 to month 15). The transformed scores were used to compute effect size. Paired t-tests were used to test for change within groups and independent groups t-tests were used to test differences between groups. Drinking data were incomplete for a number of the participants in the 0 and 1 session treatment groups. The highest level of missing data occurred at week one, where data were available for 57% of the 0 session group and 73% of the 1 session group. By follow-up, data were available for about 80% of participants in both these dropout groups.

## Results

Table 2 compares our results to the Project MATCH's published results [20] on the correlations between number of treatment sessions attended (0 to 12, or 0 to 4 for MET) and the two primary outcome measures, drinks per drinking day and percent days abstinent. The table shows that our results are essentially identical to the official results. This indicates that the data used in the present study are correct. Additionally the results show rather low correlations between number of treatment sessions attended and outcome, particularly long term outcome. The percent of variance explained can be obtained by squaring the correlation coefficient. The amount of outcome that can be attributed to attending treatment ranges from 0 to 9%, with a median of approximately 3%.

**Table 2:** A comparison of our results to the results published by the official MATCH study group shows correlations between number of treatment sessions attended and drinking outcome.

Percent Days Abstinent						
	Our results			Published MATCH results		

Month	CBT*	MET	TSF	CBT	MET	TSF

6	.2556	.0269	.2678	.26	.03	.27
9	.1743	.0135	.2666	.17	.01	.27
12	.1685	.0011	.2657	.17	.00	.27
15	.1111	.0583	.2162	.11	.06	.22
						
Drinks per drinking day						

Month	CBT	MET	TSF	CBT	MET	TSF

6	-.2281	-.0969	-.2832	-.22	-.10	-.28
9	-.2112	-.0856	-.2875	-.21	-.09	-.29
12	-.1372	-.0425	-.3028	-.14	-.04	-.30
15	-.1257	-.0795	-.2073	-.13	-.08	-.21

* CBT = Cognitive Behavioral Therapy

MET = Motivational Enhancement Therapy

TSF = Twelve Step Facilitation Therapy

Table 3 displays the same type of correlations as in Table 2, but this time the drinking measures were for the weeks during treatment. The table shows that there is a relationship between drinking level at these early time points and number of treatment sessions. Note that drinking level at week one predicts the total number of weeks the participant will remain in treatment.

**Table 3:** Correlations between number of treatment sessions attended and drinking during the course of the study.

	Percent Days Abstinent			Drinks Per Drinking Day		
Week	CBT*	MET	TSF	CBT	MET	TSF

1	.207	.107	.278	-.232	-.150	-.251
2	.279	.131	.301	-.311	-.183	-.242
3	.302	.115	.373	-.340	-.152	-.309
4	.342	.108	.375	-.359	-.131	-.303
5	.356	.157	.407	-.361	-.183	-.344
6	.371	.165	.373	-.346	-.156	-.304
7	.354	.184	.391	-.358	-.209	-.346
8	.345	.181	.375	-.326	-.203	-.326
9	.352	.140	.397	-.339	-.175	-.375
10	.400	.149	.378	-.384	-.154	-.331
11	.399	.136	.418	-.432	-.156	-.395
12	.395	.121	.436	-.404	-.160	-.413

* CBT = Cognitive Behavioral Therapy

MET = Motivational Enhancement Therapy

TSF = Twelve Step Facilitation Therapy

Tables 4 and 5 display the mean follow-up data for the two primary drinking outcomes (percent days abstinent and drinks per drinking day) of Project MATCH. Outcome at follow-up is a mean of the data from 4 to 15 months. Overall the data show that the three treatments were fairly equal and that patients who attended more sessions had somewhat better outcomes. However, there was one anomalous group. Those who dropped out after one session (the 1 treatment group) had worse outcomes than those who dropped out before attending even one session. They had worse scores than this zero treatment group on 46 of 50 measures (percent days abstinent and drinks per drinking day at pre-treatment, 12 weeks of scheduled therapy, and 12 follow-up time points). All the data are shown in the attached file [see Additional file 1]. They also had worse scores than the other treatment groups (those who attended 2 to 12 sessions) on 526 of 550 measures. The one treatment group was significantly worse that the zero treatment group over all time points for both percent days abstinent (F = 4.73 (1, 130) p = .031) and drinks per drinking day (F = 4.51 (1, 130) p = .036).

**Table 4:** Percent days abstinent at follow-up.

Total Number of Sessions Participant Received Therapy	Cognitive Behavioral (CBT) %	Twelve-Step Facilitation (TSF) %	Motivation Enhancement (MET) %	Mean of All
0	.68	.69	.75	.72
1	.64	.61	.70	.64
2	.74	.69	.73	*
3	.68	.63	.72	*
4	.50	.76	.77	*
5	.79	.69		.72
6	.78	.82		.80
7	.73	.72		.73
8	.77	.63		.70
9	.77	.78		.78
10	.74	.86		.80
11	.83	.85		.84
12	.85	.87		.86

Note: Means were calculated for total sample available at follow-up.

* Not calculated since MET was restricted to 4 sessions

**Table 5:** Drinks per drinking day at follow-up.

Total Number of Sessions Participant Received Therapy	Cognitive Behavioral (CBT) %	Twelve-Step Facilitation (TSF) %	Motivation Enhancement (MET) %	Mean of All
0	6.54	7.42	7.71	7.32
1	8.22	8.66	5.98	7.84
2	6.03	6.55	6.91	*
3	9.25	7.37	5.96	*
4	7.74	4.78	4.71	*
5	3.78	6.62		5.65
6	7.30	4.19		5.51
7	5.90	6.50		6.19
8	4.70	6.00		5.35
9	5.04	5.69		5.40
10	6.14	3.72		4.95
11	4.50	2.97		3.78
12	3.23	3.18		3.21

Note: Means were calculated for total sample available at follow-up.

* Not calculated since MET was restricted to 4 sessions

Next, in order to better illustrate what is happening in these data, mean drinking levels of participants who dropped out before receiving any treatment (N = 100 at baseline) is compared to those who attended all 12 sessions of Cognitive Behavioral or Twelve-Step Therapy (N = 355 at baseline). Also included are the anomalous group identified above, i.e., the group that dropped out after attending only one treatment session (N = 121 at baseline). We can dispense with displaying the results for groups 2 through 11 without losing any information because of the linear relationship between number of treatment sessions and drinking level shown in Tables 2 and 3. Scores for these other groups, who attended between 2 and 11 treatment sessions, fell in a linear manner between the 0 and the 12 session groups (as shown in Tables 4 and 5 and the Additional File 1).

There was no relationship between membership in the groups (0, 1 or 12) and inpatient or outpatient status (Chi square = .06 (2) p = .966) or gender (Chi square = .43 (2) p = .805). However there was a strong relationship with treatment site (Chi square = 93.90 (20) p < .0001). Some sites had larger number of participants dropping out before treatment, some had larger numbers dropping out after one treatment, and some had larger numbers attending the full 12 sessions. Some of this difference may have been due to different procedures at different sites, some may have been due to characteristics of the therapists or participants.

**Figure 1:**
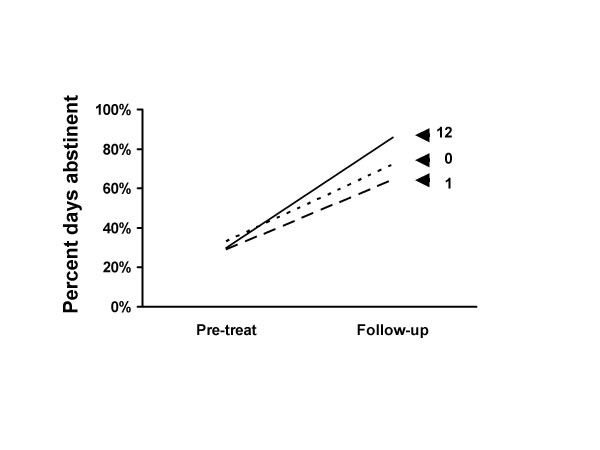
Percent days abstinent at follow-up. Percent days abstinent at pre-treatment and follow-up for patients who received 0, 1 or 12 treatment sessions. The 0 treatment dropout group showed great improvement from pre-treatment to follow-up. The 1 session attendance dropout group had worse scores than did the 0 treatment group. These data suggest that most of the improvement in the full 12 session attendance group cannot be due to treatment. See also Figure 2.

**Figure 2:**
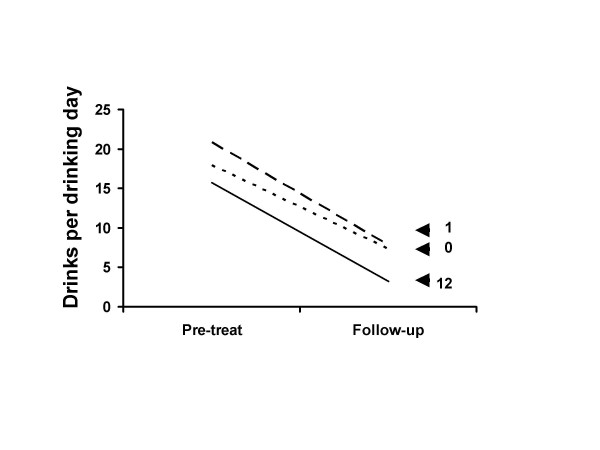
Drinks per drinking day at follow-up. Drinks per drinking day at pre-treatment and follow-up for patients who received 0, 1 or 12 treatment sessions. The 0 treatment dropout group showed great improvement from pre-treatment to follow-up. The 1 session attendance dropout group had worse scores than did the 0 treatment group. These data suggest that most of the improvement in the full 12 session attendance group cannot be due to treatment. See also Figure 1.

Figures 1 and 2 show pre-treatment and follow-up outcome data for the 0, 1 and 12 session groups. The figures shows large improvements in percent days abstinent (Figure 1) and drinks per drinking day (Figure 2) for participants who attended either 0, 1 or all 12 treatment sessions. The improvement in both measures is highly significant for all groups (Table 6). Participants who received either 0, 1 or 12 treatment sessions are displayed. Shown are the raw score means and the statistical tests and effect size estimates which were calculated using the Project MATCH transformed scores. Participants in the 0 and 1 treatment session groups dropped out and received little or no treatment.

**Table 6:** Change in primary outcomes for various periods of the study for participants who dropped out early (0 or 1 week) and for those who received full treatment (12 weeks).

Total Improvement								
	Percent days abstinent							

		Group	Pretreatment	Follow-up	t=	df	p=	Effect

		0	33	72	1.95	85	.000	1.498
		1	29	64	9.30	105	.000	1.320
		12	30	86	31.74	354	.000	2.042

	Drinks per drinking day							

		0	17.9	7.3	1.75	85	.000	1.534
		1	22.8	7.6	11.99	105	.000	1.552
		12	15.7	3.2	34.68	354	.000	2.494

Instantaneous Improvement (week 1)								

	Percent days abstinent							

		Group	Pretreatment	Week 1	t=	df	p=	Effect

		0	32	81	9.17	56	.000	1.981
		1	30	69	8.43	87	.000	1.447
		12	30	92	35.30	339	.000	2.317

	Drinks per drinking day							

		0	18.7	3.8	1.75	56	.000	2.097
		1	22.5	8.7	9.69	87	.000	1.770
		12	15.6	1.5	35.28	339	.000	2.909

Acute Treatment Phase (weeks 1 & 12)								

	Percent days abstinent							

		Group	Week 1	Week 12	t=	df	p=	Effect

		0	81	81	.19	56	.852	-.025
		1	69	63	1.69	87	.098	-.174
		12	92	96	2.82	339	.005	.155

	Drinks per drinking day							

		0	3.8	5.0	1.08	56	.283	-.172
		1	8.7	8.8	.82	87	.415	-.090
		12	1.5	1.1	2.05	339	.041	.126

The participants who dropped out after one session (the 1 session group) had worse scores than the other groups at pre-treatment as well as follow-up on both measures. Additionally, at baseline the 0 treatment group's scores were noticeably worse than the 12 treatment group's scores on drinks per drinking day (t = 3.26, 395 df, p = .001).

Effect sizes for all groups are also shown in Table 6. For percent days abstinence, those who dropped out before receiving any treatment had an effect size of 1.50, and those who attended all 12 sessions had an effect size of 2.04. For drinks per drinking day, there were effect sizes of 1.53 for the zero treatment group and 2.49 for the full treatment group. The estimated no-treatment effect sizes as proportions of the full treatment response was .73 (1.50/2.04) for percent days abstinent and .61 (1.53/2.49) for drinks per drinking day.

**Figure 3:**
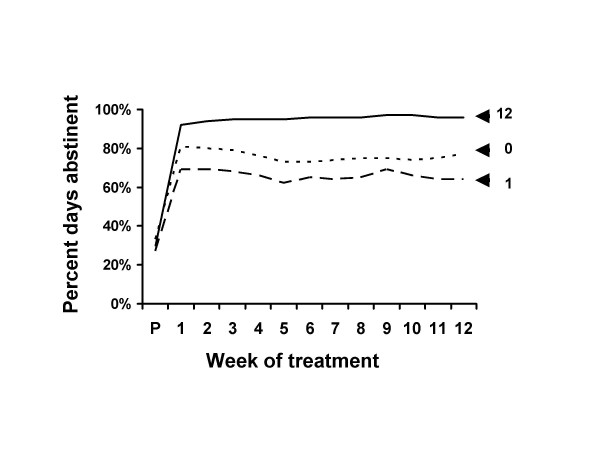
Percent days abstinent during treatment. Percent days abstinent at pre-treatment and during the 12 weeks of treatment for patients who received 0, 1 or 12 treatment sessions. The effective improvement in drinking was instantaneous, evident at week 1. The improvement was maintained at the same approximate level for the 12 weeks of scheduled treatment. The effect occurred for all groups, whether they attended all 12 treatment sessions, only one treatment session, or did not attend any treatment sessions. See also Figure 4.

**Figure 4:**
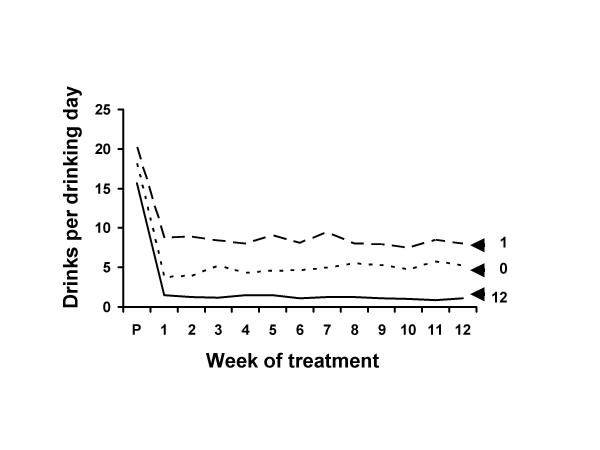
Drinks per drinking day during treatment. Drinks per drinking day at pre-treatment and during the 12 weeks of treatment for patients who received 0, 1 or 12 treatment sessions. The effective improvement in drinking was instantaneous, evident at week 1. The improvement was maintained at the same approximate level for the 12 weeks of scheduled treatment. The effect occurred for all groups, whether they attended all 12 treatment sessions, only one treatment session, or did not attend any treatment sessions. See also Figure 3.

Figures 3 and 4 show the data for the 12 weeks of the treatment period. Improvement from pre-treatment to week 1 was statistically highly significant for all groups for both measures (Table 6). The estimated no-treatment effect sizes as proportions of the full treatment response was .85 (1.98/2.32) for percent days abstinent and .72 (2.10/2.91) for drinks per drinking day. Only the 12 session group showed significant improvement from week 1 to week 12 (Table 6).

Change from week one to follow-up showed deterioration for the zero and full treatment groups for both measures. There were no significant differences between the zero treatment dropout group and the full treatment group from week 1 to follow up in percent days abstinent (t = .25, 395 df, p = .800) or in drinks per drinking day (t = 1.22, 395 df, p = .222).

## Discussion

The results suggest that treatment was not particularly effective. The following lines of evidence point to this conclusion. Correlations between treatment attendance and outcome were very small (as shown in Table 2). A median 3% of the variance in outcome might be attributed to treatment.

The correlations existed before most treatment occurred, at week 1 (Table 3). We would normally infer from the correlations in Table 2 that more treatment produces better drinking outcomes, but the Table 3 correlations suggests the reverse, that better drinking levels predict more treatment.

Nearly two thirds of the long term improvement in the full treatment group was matched by the untreated rapid dropout group (Figures 1 &2 and Table 6). Only in the remaining one third could there be a subcomponent consisting of a treatment effect.

Most of the improvement was instantaneous, occurring at week 1, before the participants had received the bulk of their treatment (Figures 3 &4 and Table 6). Although the full treatment group received 11 more therapy sessions, the additional improvement was of small magnitude. For example, at week one percent days abstinence had increased by over 60%, and the additional 11 weeks of treatment increased it by only 4%. If treatment were the causal agent we would expect that the effect would occur over the course of weeks with the administration of treatment.

There was a similar instantaneous improvement in untreated alcoholics (Figures 3 &4 and Table 6). The effect size estimates suggested that nearly three fourths of the instantaneous improvement in the full treatment group was matched by the untreated group.

Those who received zero treatment sessions had better outcomes than those who received one session (Figures 1 to 4 and Tables 4 to 6). The implication of this is discussed below.

Improvement was maintained over time even in the no treatment group (Figures 1 to 4 and Table 6). Change from week 1 to follow-up was not significantly different between the zero and full treatment groups. In both groups the week 1 to week 12 improvement was lost by follow-up. These data do not support the contention that retention of clients in treatment for as long as possible increases the chances that they will derive benefit from therapy.

A more reasonable interpretation of these data is that they illustrate the importance of selection effects, i.e., participants who reduce their alcohol consumption are more likely to enter or remain in treatment and those who continue drinking are more likely to drop out of treatment. One of the best studies of alcoholism treatment outcome was conducted by the Rand Corporation in the late 1970s [10]. Participants were patients who attended inpatient or outpatient treatment at centers across the United States. They found that "it is possible that the correlation [between attendance and outcome] arises from selection effects, such that the better motivated or more successful patients continue in treatment, whereas the more intractable cases drop out. Such a pattern could result from subject self-selection or from the operation of the treatment environment in encouraging continued participation for more responsive patients." (page 155). The likely selection effect in the current data was illustrated by the anomalous group participants who dropped out after attending only one treatment session as shown in Figures 1 and 2. Those who received zero treatment had better outcomes than those who received one session of treatment. Few would argue that this shows that treatment was harmful. A more likely explanation for this difference is some sort of self-selection. The higher drinking level of the 1 session dropout participants at baseline suggests that they may have been more dependent on alcohol than those in the 0 session dropout group. The relative higher level of dependence may have put these individuals under more pressure to do something about their drinking, explaining why they did not drop out prior to the first session. A similar logic could apply to the outcomes of the consistent attendees of the full treatment group. The likely selection effect is also shown in that participants with higher drinking levels at week 1 were more likely to drop out of treatment (Table 3).

The decreased drinking in both untreated and treated participants can be explained by a number of factors. One factor is that part of the effect is not real; many active alcoholics underreport drinking. Collateral informant interviews and other verification techniques are only partially effective in correcting the data. The Rand study [10], for example, found that 30% of the collateral informants were unable to provide information. Underreporting can make treatment appear more effective than it actually is.

Additionally, there are a number of non-treatment effects likely to result in reduced drinking [19,21-23]. In order to enter the trial participants had to first achieve a level of abstinence or reduced intake. If a participant arrives at a site in an intoxicated state immediate action is required by staff, such as admission to a detox unit, or detainment in the waiting room until the breath alcohol level returns to normal. These rules would have applied to each participant in Project MATCH at the time of enrollment and would have contributed to the rapid improvement seen in the week one data. The pre-study screening procedures used in clinical trials, both the overt criteria and the subjective criteria, are designed to select participants who are motivated to reduce their drinking. Enrolling in the trial suggests that the alcoholic has crystallized a decision to reduce or abstain from drinking. Once in the trial, the continued monitoring of drinking behavior by staff personnel may have both motivational and therapeutic benefits. For example, in one study with a 2 year follow-up [21], over half the participants indicated they liked the "caring, concern and help" follow-up telephone contact, and in another [24], the telephone interviewers reported that they usually entered in a sympathetic interaction with the study participants. Such positive empathetic contact could be of therapeutic benefit.

There are a number of limitations to these analyses. The data from two thirds of the subjects were not used in the illustration of mean drinking levels shown in the figures and in Table 6. However these data were used in Tables 2 though 5, and are presented in more detail in the attached data [see Additional File 1]. The linear relationship shown in Tables 2 and 3, and the means in Tables 4 and 5 indicated that no information was left out. Additionally, other analyses of these groups have been previously published, e.g., by the Project MATCH research group [20]. We chose to highlight several specific groups. The logic of selecting the group that received no treatment and the group that received all 12 sessions of treatment was clear – they offered an unambiguous treatment comparison. The data presented here show that the outcomes of the 12 session group were better than the outcomes of participants who received between 2 and 11 sessions, making the 12 session group a fair comparison. Additionally we identified an anomalous group, the one session rapid dropouts, and used that group in an attempt to interpret the data. The mathematical bases for the anomalous designation, and thus the selection of this group, were presented.

Analyses were primarily limited to descriptive and simple inferential statistics. This was done because the findings are likely to be extremely controversial. We have therefore presented results that are easily replicated, and easily understood.

Although the results are essentially negative, suggesting that current treatments are not effective, we do not offer suggestions for future directions. We feel we will have made a contribution if the data presented can be accepted as accurate. If they are accepted then implications for future research and treatment will naturally follow. For example, if the patient's motivations, opportunities, beliefs and hopes are the critical issues, how do we measure them? How do we influence them? How do they interact with the treatment environment?

It may be that pre-treatment patient characteristics (e.g., level of dependence, social support, etc.) have a large influence on both the number of treatment sessions attended and drinking outcome. However, even if this is true, it would not be evidence of treatment effectiveness. Only if one could show that positive prognostic factors were weighted heavily against the treatment attendees and in favor of the dropouts would these results be open to reinterpretation. The baseline and week one drinking data presented here do not support the likelihood of such a possibility. Additionally, there is no evidence in the literature to support the notion that, for example, alcoholics who lack social support are more likely to enter or remain in treatment. There are a large number of both positive and negative reasons why alcoholic participants drop out of clinical trials. Positive reasons include work commitments, pregnancy, re-location to another area and remission from drinking. Negative reasons include continued or increased drinking, abuse of other substances, attitude towards the clinical staff or environment, physical illness, hospitalization and incarceration.

Conclusions drawn from therapy delivered in clinical trials might not be applicable to therapy in other settings. We might well expect great differences in clinical effectiveness between different therapists, and between different treatment programs. However it can be argued that the large non-treatment effect seen in this study is present in other aggregated outcome studies published in the literatures. Miller and others [9] presented results from a number of such trials, in addition to Project MATCH. Table 1 summarized their findings for the two outcome variables studied here. The outcomes of the different studies are remarkably similar. The similarity in results would suggest that the non-treatment effect identified here may be present in all these studies.

The outcome variables in these analyses were the original primary MATCH outcome variables. We have been able to show that the analyses of these variables, and the treatment attendance variable, are in perfect concordance with published analyses of the Project MATCH Research group [20]. Over 60 publications have been generated by Project MATCH, but, to the best of our knowledge, all have overlooked the main finding of this study, i.e., the good outcomes of the zero treatment group when compared to the full treatment group and that the improvement in all groups occurred immediately after enrollment in the trial. Ineffective treatment would be the most parsimonious explanation for the rather surprising main findings of Project MATCH, that there was no match between patient characteristics and different types of treatment, and that all three treatments were equal.

There may be similarities between these results, for alcoholism patients, and effects seen in some other types of patients. Depressed patients sometimes report significant improvement after enrolling in clinical trials but before receiving therapy [25]. Recent time-course analyses in depression report sudden decreases in depression regardless of treatment condition [26]. These rapid responders were associated with better outcome at the end of the treatment and into follow-up [27].

It is difficult to compare the high quality follow-up data of Project MATCH to that in the alcoholism literature, much of which are collected under quite different circumstances. The zero treatment participants at the final follow-up interval (month 15) reported a mean of 25.1 drinks per week, with 45% (35/78) abstinent. These outcomes appear somewhat better than those recently summarized in the literature [17]. Of some 17 studies than included placebo or no treatment conditions, with and without prior detoxification, a mean (for studies) was 21% abstinent, and the average participant was drinking 31 drinks per week [17].

Exaggerated claims of treatment effectiveness can have undesirable consequences for patients, for therapists, and for science. Patients who fail an "effective" treatment may feel even more hopeless. This increased despair may be extremely deleterious in people with such life-threatening habits. Therapists may feel inadequate or frustrated with repeated failures. For science, exaggerated claims tend to shift focus into unproductive directions, and to obscure the pertinent facts that are necessary in order to move the science forward.

While this study shows that three of the best treatments currently available for addiction were not very effective, it remains likely that many severely dependent alcoholic individuals benefit from external help. By suggesting practical and helpful ways for dealing with the problems of addiction, therapy may help a patient regain a sense of control over his or her life. We are not suggesting that alcoholism treatment should be discontinued or even reduced. People with alcohol problems clearly need all the help our society can give them.

## Conclusion

These results suggest that current psychosocial treatments for alcoholism are not particularly effective. The improvements in drinking appear to be due to selection effects. Alcoholics who decide to enter treatment are likely to reduce drinking. Those who decrease their drinking are more likely to remain in treatment. Widespread acceptance of these results would have a profound influence on alcoholism research and treatment because it would shift focus away from treatment components and toward patient characteristics and beliefs.

## Competing interests

The author(s) declare that they have no competing interests.

## Authors' contributions

RBC conceived of the study, participated in drafting the manuscript and performed the statistical analysis. DAF participated in drafting the manuscript. All authors read and approved the final manuscript.

## Pre-publication history

The pre-publication history for this paper can be accessed here:



## Supplementary Material

Additional File 1 Drinking outcomes for all groups at each time point. Shown are mean percent days abstinent and drinks per drinking day for all participants in the Project MATCH data set. Participants were categorized by number of treatment sessions attended. Participants in MET are not included in the 2, 3 and 4 treatment session means. Click here for file
